# Measuring Anti-aging Effects in *Drosophila*

**DOI:** 10.21769/BioProtoc.5305

**Published:** 2025-05-05

**Authors:** Hyun-Jin Na, Joung-Sun Park

**Affiliations:** 1 Aging and Metabolism Research Group, Division of Food Functionality Research, Korea Food Research Institute, Wanju, Korea; 2 Institute of Nanobio Convergence, Pusan National University, Busan, Korea; 3 Department of Molecular Biology, Pusan National University, Busan, Korea

**Keywords:** *Drosophila*, Anti-aging, Vitamin D, Vitamin D receptor, Aging marker, DNA damage, Abnormal centrosome amplification, Intestinal stem cell

## Abstract

One of the major factors contributing to aging and age-related diseases is the well-understood decline in the function of adult stem cells. Quantifying the degree of aging in adult stem cells is essential for advancing anti-aging mechanisms and developing anti-aging agents. However, no systematic approach to this exists. In this study, we developed a method to quantitatively assess the degree of aging in adult intestinal stem cells using a *Drosophila* midgut model and two aging markers. First, aging was induced in *Drosophila* with the desired genotype, and the anti-aging agent was administered 7 days before dissection. Then, the levels of two intestinal stem cell aging markers found in *Drosophila* (PH3 and γ-tubulin) were measured using immunohistochemistry. Finally, fluorescence microscopy was employed to count the number of aging markers and take images, which were analyzed using image analysis software. Using this approach, we quantitatively analyzed the effects of anti-aging agents on the aging of adult intestinal stem cells. This methodology is expected to significantly expedite the development of anti-aging agents and substantially reduce the research costs associated with aging-related studies.

Key features

• PH3 and γ-tubulin serve as reliable markers for quantitatively assessing aging in *Drosophila* intestinal stem cells.

• This method for discovering anti-aging agents involves processes such as aging induction, treatment with anti-aging agents, dissection, fixation, antibody staining, and analysis of the results.

• Vitamin D, similar to metformin and β-hydroxybutyrate, is an anti-aging agent.

• Quantitative analysis of adult stem cell aging will enable the rapid and accurate identification of anti-aging agents and efficacy validation.

## Graphical overview


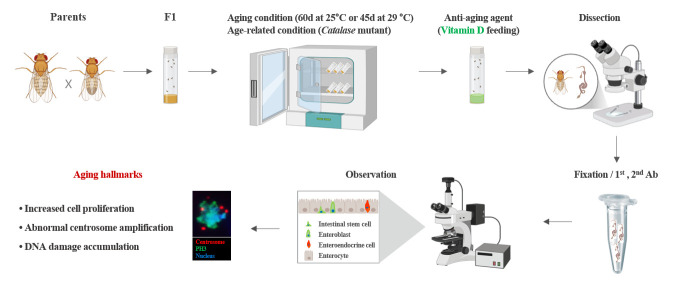


**Experimental setup and procedure for measuring anti-aging effects**

## Background

Adult stem cells play a key role in tissue homeostasis and regeneration because of their ability to self-renew and generate differentiated cells. Age-related changes in these stem cells are closely associated with aging and age-related tissue diseases [1,2]. Many studies have aimed to identify changes in adult stem cells related to aging and develop anti-aging agents to slow the aging process [3–8]. Although studies have shown that various factors contribute to aging, there are limitations to quantifying these factors. Currently, there is no statistical method for quantifying adult stem cell aging.

First, a suitable model system is required to quantitatively study adult stem cell aging. *Drosophila*, due to their short life span and genetically modifiable midgut function, are a widely utilized model organism for aging research, encompassing investigations of adult stem cells and their microenvironmental as well as changes associated with aging [2,8,9]. In the adult *Drosophila* midgut, intestinal stem cells (ISCs), marked by delta (Dl), are the only proliferation cells that give rise to two distinct differentiated progenies: enterocytes (ECs), which arise from enteroblasts (EBs) induced by strong Notch (N) signaling, and enteroendocrine (EE) cells, which are derived from EBs activated by weak N signaling [10]. These four types can be identified using specific markers such as Dl (ISCs), phospho-histone H3 (PH3, dividing ISCs), esg-GFP (ISCs and EBs), Su-GFP (EBs), Pdm and Myo-GFP (ECs), and Prospero (Pros) and Pros-GFP (EE cells) [7–8,10].

Second, utilizing appropriate markers is crucial for quantitatively assessing ISC aging. Using an anti-PH3 antibody, Choi et al. revealed age-related increases in *Drosophila* ISC division, which was corroborated by subsequent studies [3,11,12–16]. The anti-PH3 antibody specifically binds to phospho-histone H3 at serine 10, a modification that occurs during mitosis; therefore, it is widely used as a marker for identifying mitotic cells [14]. Using an anti-γH2AX antibody, Park et al. observed age-related increases in DNA damage accumulation in ISCs [17]. The anti-γH2AX antibody specifically recognizes and binds to phosphorylated histone H2AvD at serine 137, a modification analogous to the γH2AX phosphorylation in mammals [17]. Using an anti-γ-tubulin antibody, Park et al. revealed that age-related increases in abnormal centrosome amplification occur in ISCs and renal stem cells (RSCs) [14,15,18]. The anti-γ-tubulin antibody specifically recognizes and binds to γ-tubulin, a key protein in microtubule nucleation and organization in the division of eukaryotic cells [14,15].

Third, sufficient aging and age-related conditions are required to quantitatively study adult stem cell aging. It is well established that 60-day-old flies maintained at 25 °C and 45-day-old flies maintained at 29 °C serve as effective models for aging when compared to young flies [14–17]. In addition, the model of intrinsic oxidative stress *Catalase* heterozygous mutant flies is a widely used age-related model [14,18].

Fourth, appropriate anti-aging agents are essential for quantitatively assessing their effects on adult ISCs. Previous studies have reported that metformin, a commonly prescribed medication for the management of type 2 diabetes, and β-hydroxybutyrate, a type of ketone body produced during periods of fasting, prolonged exercise, or low-carbohydrate diets, exhibit anti-aging properties [15,19]. In recent studies, age-related declines in Vitamin D (VitD) synthesis and VitD receptor (VDR) expression have been associated with age-related diseases [16]. However, few studies have explored the role of VitD/VDR in adult stem cells, highlighting the need for further related investigations. In this study, by utilizing the age markers of *Drosophila* ISCs and age-related conditions, we aimed to provide a detailed and precise method for the quantitative measurement of adult stem cell aging. This method can be used for the quantitative analysis of anti-aging agent development and efficacy research, which will contribute to reducing research costs and shortening the research period spent in anti-aging fields in the future.

## Materials and reagents

**Biological materials**

1. *Oregon-R* (Bloomington Drosophila Stock Center, BDSC, Bloomington, IN, USA, #5)

2. *Catalase* heterozygous mutant flies (*Cat^n1^* mutant) (BDSC, catalog number: 4014) [20]

3. *UAS-hr96-RNAi* lines (Vienna Drosophila RNAi Center, VDRC, Vienna, Austria, catalog number: 330288, catalog number: 10958)

4. *Myo1A-Gal80^ts^* flies, obtained from B.A. Edgar

5. Rat anti-GFP antibody (Nacalai Tesque Inc., Kyoto, Japan, catalog number: 04404-84)

6. Rabbit anti-phospho-histone H3 (PH3) (Millipore, Billerica, MA, USA, catalog number: 06-570)

7. Mouse anti-γ-tubulin (Sigma-Aldrich, St. Louis, MO, USA, catalog number: T6557-.2ML)

8. Goat anti-rat FITC antibody (Jackson ImmunoResearch, catalog number: 415-095-166)

9. Goat anti-rabbit Alexa Fluor^®^ 647 antibody (Jackson ImmunoResearch, catalog number: 305-605-003)

10. Goat anti-mouse Cy3 antibody (Jackson ImmunoResearch, catalog number: 205-165-108)

**Reagents**

1. EM-grade paraformaldehyde 16% aqueous solution (Electron Microscopy Science, catalog number: 15710)

2. 10× phosphate-buffered saline (PBS) (Ambion, catalog number: AM9624)

3. Methanol (Sigma-Aldrich, CAS number: 67-56-1)

4. 10% Triton X-100 (Millipore, CAS number: 9036-19-5)

5. 4′,6-diamidino-2-phenylindole (DAPI) (Molecular Probes, catalog number: D1306)

6. Vectashield (Vector Laboratories, catalog number: H-1000-10)

7. 1α,25-Dihydroxyvitamin D3, D1530 (VitD) (Sigma-Aldrich, catalog number: D1530)

8. Ultra-pure water (Welgene Inc., catalog number: ML 019-02)

9. Propionic acid (Junsei Chemical Co., CAS number 79-09-4)

10. Butyl 4-hydroxybenzoate (Junsei Chemical Co., Ltd., CAS number 94-26-8)

**Solutions**

1. Standard food (see Recipes)

2. Vitamin D food (VitD) (see Recipes)

3. Bokinin (see Recipes)

**Recipes**

**1. Standard food**

**Table:** 

Reagent	Final concentration	Quantity or Volume
Sucrose	10% (w/v)	100 g
Cornmeal	7% (w/v)	70 g
Yeast	2% (w/v)	20 g
Agar	1% (w/v)	10 g
Propionic acid	0.5% (v/v)	5 mL
Bokinin	0.3% (v/v)	3 mL
Water	79.2% (v/v)	1 L

**2. Vitamin D food (VitD)**

**Table:** 

Reagent	Final concentration	Quantity or Volume
10 mM Vitamin D	100 nM (v/v)	0.1 mL
Standard food	n/a	9.9 mL
Total	n/a	10 mL

**3. Bokinin**

**Table:** 

Reagent	Final concentration	Quantity or Volume
Butyl 4-hydroxybenzoate	10% (v/v)	10 g
Ethanol	n/a	70 mL
Water	n/a	30 mL

**Laboratory supplies**

1. Microtube (Axygen, model: MCT-175-X)

2. Microscopic slides (Paul Marienfeld GmbH & CoKG, model: 31593)

3. Microscope cover glasses (24 × 32 mm, Deckglaser, model: HSU-0101172)

4. Tip 20 μL (Axygen, model: TF-20-RSm TF-200-R-S, TF-1000-R-S)

5. Vonetex latex gloves (Hartalega Sdn. Bhd.)

## Equipment

1. Microscope (Carl Zeiss Inc., model: Axioskop 2 Plus)

2. Aerobic incubator (Sanyo, model: MIR-254-PK)

3. Sigma Plot 14.5 (Systat Software Inc., San Jose, CA, USA)

4. Stereomicroscope with Axiocam 208 color camera (Zeiss, model: Stemi 508)

5. Fluorescent microscope with AxioCam MRm (Zeiss, model: Axioplan2)

6. Precision tweezer (Techni-Tool Tweezer Ni-Cr-Mo Superalloy, Type 5, Techni-Tool, Worcester, PA; catalog number: 33-04594-01)

7. Single orbital shaker (Daeil biotech., model: PM-6249)

## Software and datasets

1. AxioVision Rel. 4.8 software (Carl Zeiss Inc.)

2. SigmaPlot 14.5 package (Systat, Software Inc., San Jose, California, USA)

## Procedure

Below, we describe a step-by-step procedure for measuring the effects of anti-aging agents in adult intestinal stem cells using *Drosophila* midgut with temperature-controlled gene expression, immunochemistry, and feeding assays. All washing steps throughout the protocol are conducted with 800 μL unless stated differently.

**A. Fly collection**

1. Stock culture conditions: All *Drosophila* fly stocks were reared at 25 °C and provided with a standard cornmeal-molasses diet under a 12/12 light/dark (L:D) cycle. To avoid larval overpopulation in all vials, 50–60 adult flies per vial were transferred to new food vials every 2–3 d during their lifetime.

2. Genotypes of the flies used:

a. *Myo^ts^>GFP* flies were obtained by crossing *Myo1A-GAL4/CyO;UAS-GFP,tub-Gal80^ts^/TM6B (Myo^ts^*) females and *Oregon-R* males.

b. *Myo^ts^>GFP+Cat^n1^* flies were obtained by crossing *Myo^ts^* females and *Cat^n1^* mutant males.

c. *Myo^ts^>GFP+hr96Ri* flies were obtained by crossing *Myo^ts^* females and *UAS-hr96Ri* (#330288 or #10958) males.

The Gal80ts technique was used for *hr96* knockdown, an orthologous to human VDR, at specific developmental stages and tissues [21]. The experimental flies were housed at 22 °C until adulthood. For the collection of young adults, we used 1–3-day-old flies that were transferred to an environment at 29 °C for 45 days to activate the GAL4/Gal80 system, and UAS-RNAi was used for gene knockdown. For more details regarding the GAL4/GAL80 system, see [22,23]. The results described herein were obtained using female flies.

**B. Aging and age-related condition induction**

1. Aging conditions: Transfer 1-day-old *Myo^ts^>GFP* flies to 29 °C for 45 days to induce aging.

2. Age-related condition: Transfer 1-day-old *Myo^ts^>GFP+Cat^n1^* and *Myo^ts^>GFP+hr96Ri* flies to 29 °C for 10 days to induce age-related conditions (*Catalase* mutant).

**C. Vitamin D feeding**

**Figure 1.**
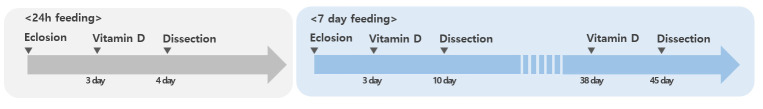
Process of administering vitamin D. Three-day-old *Myo^ts^>GFP, Myo^ts^>GFP+Cat^n1^*, and *Myo^ts^>GFP+hr96Ri* or 38-day-old *Myo^ts^>GFP* flies were treated with 100 nM VitD in standard food for 7 days at 29 °C in the dark.

To measure the anti-aging effects of VitD, treat 3-day-old *Myo^ts^>GFP, Myo^ts^>GFP+Cat^n1^*, and *Myo^ts^>GFP+hr96Ri* or 38-day-old *Myo^ts^>GFP* flies with 100 nM VitD [16,24] in standard food media for 7 days at 29 °C in the dark (Figure 1). Transfer the flies to new food vials every two days.

**D. Gut dissection**

**Figure 2.**
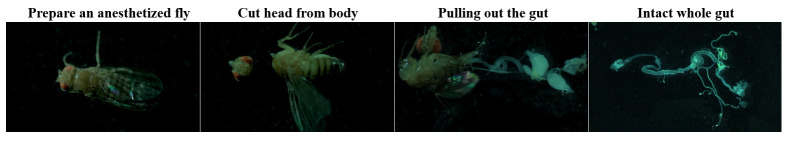
Whole gut dissection for immunostaining. Ten-day-old *Myo^ts^>GFP, Myo^ts^>GFP+Cat^n1^*, and *Myo^ts^>GFP+hr96Ri* or 45-day-old *Myo^ts^>GFP* flies were dissected using forceps in a hole slide glass with 1× PBS. Ten entire intact guts were placed in a microtube (1.5 mL) containing 1×PBS on ice, followed by immediate fixation.

To dissect the intact whole gut for immunostaining, anesthetize adult flies using carbon dioxide. Carefully remove the head of the flies using anatomical forceps, slightly grab the thorax, and carefully pull the posterior end of the abdomen to take the intact midgut from the crop to the rectum (Figure 2).

**E. Fixation**

1. Dissect the entire intact adult gut on ice.

2. Fix whole guts for 30 min in 4% paraformaldehyde in 1× PBS on a single orbital shaker in the dark.

3. Subsequently, dehydrate them for 5 min in 50%, 75%, 87.5%, and 100% methanol in 4% paraformaldehyde in 1× PBS and rehydrate for 5 min in 50%, 25%, and 12.5% methanol in 1× PBST (1 × PBS + 0.1% triton X-100).

4. Then, wash them thrice for 20 min with 1× PBST.

**D. Immunochemistry**

**Figure 3.**
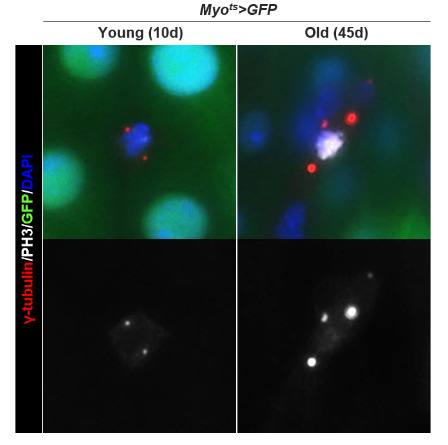
Age-related increase in centrosome amplification (CA) in *Drosophila* midgut intestinal stem cells (ISCs). The gut from 10-day-old (left panels) and 45-day-old (right panels) *Myo^ts^>GFP* were stained with anti-PH3 (white), ant-γ-tubulin (red), and anti-GFP (green) antibodies and DAPI (blue). The original magnification is 400×.

The procedure described below was used for immunostaining using anti-PH3 (1:1,000), ant-γ-tubulin (1:1,000), and anti-GFP (1:1,000) antibodies, which revealed that the number of centrosomes in dividing ISCs increased with age. Generally, two centrosomes were detected in the mitotic ISCs of 10-day-old *Myo^ts^>GFP*, but mitotic ISCs with more than two centrosomes were also observed in 45-day-old *Myo^ts^>GFP* (Figure 3).

1. Incubate samples overnight with the primary antibodies at 4 °C in 1× PBST.

2. Wash them thrice for 20 min with 1× PBST.

3. Incubate the samples at 25 °C for 1 h with the secondary antibodies and DAPI (nucleus, 1:1000) in 1× PBST.

4. Wash the guts again thrice for 20 min with 1× PBST.

5. Mount the samples using Vectashield.

6. Analyze the samples using an Axioskop 2 Plus microscope.

7. Count the number of PH3^+^ cells with centrosomes (≤2 or ≥ 3) in the whole midgut.

## Data analysis

**Figure 4.**
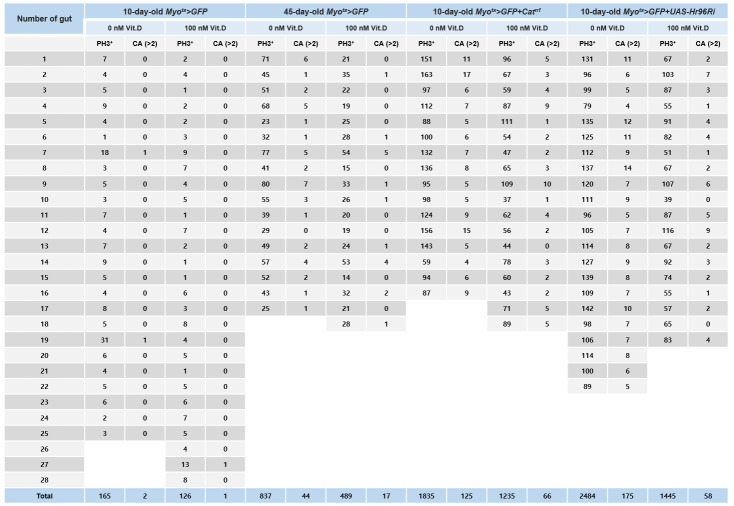
Age and age-related increases in PH3^+^ cell number and centrosome amplification (CA) in *Drosophila* midgut intestinal stem cells (ISCs). The gut from 10-day-old *Myo^ts^>GFP*, 45-day-old *Myo^ts^>GFP* (age condition), 10-day-old *Myo^ts^>GFP+Cat^n1^* (age-related condition), and 10-day-old *Myo^ts^>GFP+Hr96Ri* (age-related condition) were stained with anti-PH3 (white), ant-γ-tubulin (red), and anti-GFP (green) antibodies and DAPI (blue). Cells were counted throughout the gut for the quantitative analysis of the number of PH3^+^ cells and γ-tubulin-stained spots per PH3^+^ cell in the whole midgut.

Cells were counted throughout the gut for the quantitative analysis of PH3^+^ cells. To quantitatively analyze centrosome amplification, the number of γ-tubulin-stained spots per PH3^+^ cell in the whole midgut was determined (Table 1). We counted the number of PH3^+^ cells in the entire midgut and quantified the frequencies of these mitotic ISCs by centrosome amplification (CA, >2), as shown in Figure 4. Quantified data are expressed as the mean ± standard error. Significant differences were determined using the Student’s t-test. Sigma Plot 14.5 was used to analyze standard errors [14].

## Validation of protocol

This protocol was used to measure anti-aging effects in *Drosophila* adult intestinal stem cells in the following research articles:

- Park et al. [16]. The anti-aging effect of vitamin D and vitamin D receptor in *Drosophila* midgut. Aging (Albany NY). (Figures 3A, B and 6A–D).
- Park et al. [17]. Age and oxidative stress-induced DNA damage in *Drosophila* intestinal stem cells as marked by GammaH2AX. Exp Gerontol. (Figure 1A–C).
- Na et al. [13]. Mechanism of metformin: inhibition of DNA damage and proliferative activity in *Drosophila* midgut stem cell. Mech Ageing Dev. (Figures 1–3).
- Park et al. [14]. Increased centrosome amplification in aged stem cells of the *Drosophila* midgut. Biochem. Biophys. Res Commun. (Figures 1 and 2).
- Na et al. [15]. Metformin inhibits age-related centrosome amplification in *Drosophila* midgut stem cells through AKT/TOR pathway. Mech Ageing Dev. (Figures 1, 2B–D, and 3A, C).
- Park et al. [5]. Deficiency in DNA damage response of enterocytes accelerates intestinal stem cell aging in *Drosophila*. Aging (Albany NY). (Figure 3B).
- Park et al. [19]. Anti-Aging Effect of the Ketone Metabolite β-Hydroxybutyrate in *Drosophila* Intestinal Stem Cells. Int J Mol Sci. (Figures 1 and 2).

## General notes and troubleshooting

**General notes**

1. The *Drosophila* gut used in the experiment must be dissected intact from the crop to the rectum (including the Malpighian tubules). Even a slight wound during gut dissection can cause intestinal stem cell division, increasing the number of PH3^+^ cells throughout the gut.

2. *Drosophila* gut dissection must be performed as quickly as possible; in this study, the dissection time per experiment was within 30 min.

3. In aging studies, it is important to maintain constant adult fly growth conditions. In particular, it is important to maintain temperature, humidity, and food intake.

**Troubleshooting**

Problem 1: Weak γ-tubulin signal.

Possible causes: γ-tubulin signal is very small.

Solution: It is easier to first check the PH^+^ cells and then count the abundance of γ-tubulin in the cells at a higher microscope magnification.
